# Mental Health Issues in Madhya Pradesh: Insights from National Mental Health Survey of India 2016

**DOI:** 10.3390/healthcare7020053

**Published:** 2019-03-31

**Authors:** Arun Kokane, Abhijit Pakhare, Gopalkrishna Gururaj, Mathew Varghese, Vivek Benegal, Girish N. Rao, Banavaram Arvind, Mukesh Shukla, Arun Mitra, Kriti Yadav, Rajni Chatterji, Sukanya Ray, Akash Ranjan Singh

**Affiliations:** 1 Department of Community and Family Medicine, 2nd Floor, College Building, All India Institute of Medical Sciences (AIIMS), Saket Nagar, Bhopal 462020, Madhya Pradesh, India; Abhijit.cfm@aiimsbhopal.edu.in (A.P.); drmukeshshukla@gmail.com (M.S.); arunmitra2003@gmail.com (A.M.); drkritiyadav18@gmail.com (K.Y.); akashranjan02@gmail.com (A.R.S.); 2 Department of Epidemiology, Centre for Public Health, National Institute of Mental Health and Neuro Sciences (NIMHANS), Bengaluru 560029, Karnataka, India; epiguru@yahoo.com (G.G.); girishnrao@gmail.com (G.N.R.); aravind_baa@yahoo.co.in (B.A.); 3 Department of Psychiatry, National Institute of Mental Health and Neuro Sciences (NIMHANS), Bengaluru 560029, Karnataka, India; mat.varg@yahoo.com (M.V.); vbenegal@gmail.com (V.B.); 4 Department of Psychiatry, Bhopal Memorial Hospital and Research Centre (BMHRC), Bhopal 462038, Madhya Pradesh, India; rajnichatterji@gmail.com; 5 Department of Psychiatry, All India Institute of Medical Sciences (AIIMS), Bhopal 462020, Madhya Pradesh, India; sukanya.ray7@gmail.com

**Keywords:** mental disorder, treatment gap, health system, Madhya Pradesh

## Abstract

Background: About 14% of the global mental health burden is contributed by India. However, there exists a disparity in mental health patterns, utilization, and prioritization among various Indian states. The state of Madhya Pradesh is a low performer among Indian states, ranking lower than the national average on the Human Development Index, Hunger Index, and Gross Domestic Product (GDP). The state also performes poorly on other health-related indicators. Objectives of Study: To estimate the prevalence and patterns of mental illnesses in the state of Madhya Pradesh, India. Material and Methods: This study used the multistage, stratified, random cluster sampling technique, with selection probability proportionate to size at each stage. A total of 3240 individuals 18 years and older were interviewed. The mixed-method study that was employed had both quantitative and qualitative components. The Mini International Neuropsychiatric Interview along with 10 other instruments were used. Results: The overall weighted prevalence for any mental illness was 13.9%, with 16.7% over the lifetime. The treatment gap for all of the mental health problems is very high (91%), along with high suicidal risk and substance use in the state. Conclusions: This study provides evidence of the huge burden of mental, behavioral, and substance use disorders as well as the treatment gap in Madhya Pradesh. This information is crucial for developing an effective prevention and control strategy. The high treatment gap in the state calls for coordinated efforts from all stakeholders, including policy makers, political leaders, health care professionals, and the society at large to give mental health care its due priority. These findings also highlight the need for multi-pronged interventions rooted in health policy directed at reducing the treatment gap in the short term and disease burden in the long run.

## 1. Introduction

Mental as well as neurological and substance use disorders (MNSUDs) are becoming increasingly common, affecting 1.1 billion people (one in six individuals) worldwide and emerging as a public health problem of global concern [1,2,3]. Despite the global magnitude of mental health illness and its impact on social, economic, and human behavior being reiterated by many studies, the prioritization of mental health care is yet to be realized [3,4,5,6,7,8,9,10]. Although the mental health burden is greater in developed countries, the developing countries are showing an increasing trend in terms of both disease burden and treatment gap [1,3,11,12,13]. Almost four in five persons with mental disorder are from low- and middle-income (LAMI) countries [11].

India and China share about one-third of all the global burden in terms of disease-adjusted life years (DALYs) attributable to MNSUDs, which is greater than the combined burden of all developing countries (66 million DALYs versus 55 million DALYs). Between 1990–2003, India has seen a greater increase in the MNSUDs burden (44%) as compared to China (20%) [14]. According to the Global Burden of Disease Study 2017, mental health ranks as the leading cause for years lived with disability (YLDs) in India (22 million YLDs). The majority of this occurs in the most productive age group (15–49 years). Mental health disorder burden ranks second only to cardiovascular disease burden with 8.76% of all DALYs in the same age group. This is approximately a 30% increase in the percent of total DALY from 1990 [15].

MNSUDs categorized under the non-communicable (NCD) are recognized as important public health problems in India [16,17]. The presence of mental illness is considered as stigma, and impacts the familial, social, and occupational life of a person. This has considerable negative influence on the family and society at large. Also, there exists a disparity in health across many states in India. The constitution of India places public health in the State List, making it the state’s responsibility. States such as Kerala, Maharashtra, and Tamil Nadu perform better in health as compared to states such as Madhya Pradesh and Bihar [18]. Considering India’s overburdened health care resources and infrastructure, it is imperative to understand the magnitude and the factors associated with mental health in great detail in order to come up with an effective control strategy [19].

Madhya Pradesh is the second largest state in India, having a 7.2 million population and 51 districts [20]. It is a high-priority state for Reproductive, Maternal, Newborn, Child, and Adolescent Health (RMNCH+A) interventions for reducing maternal and child mortality. Consequently, MNSUDs have not received the needed attention from various concerned stakeholders. The insufficiency of the data from previous studies often precludes its use for the development of mental health programs in Madhya Pradesh [16,21]. Thus, in order to strengthen mental health policies and programs at the state levels, the present study was conducted as a part of the National Mental Health Survey 2015–2016 with the objective of estimating the prevalence and patterns of mental illnesses, identification of the treatment gap, health care utilization, and self-reported disability among respondents with current mental illness.

## 2. Material and Methods

The National Mental Health Survey (NMHS) of India was undertaken in 12 states of India including Madhya Pradesh during 2015–2016 [17].

### 2.1. Study Design

The community-based cross-sectional study was undertaken to assess the prevalence, pattern, treatment gap, and disability associated with mental disorders in the state. Focus group discussions were conducted to understand the perception of the community regarding the above issues. Mental health systems in the state were assessed through key informant interviews (KII) and also by collecting secondary data.

### 2.2. Study Duration

April 2015 to May 2016.

### 2.3. Sample Size

As the location of the National Institute of Mental Health and Neurosciences, Bangalore was assigned the overall responsibility of coordinating NMHS at the national level, a pilot study for NMHS was undertaken in the Kolar district of Karnataka [22]. In the pilot study, the prevalence of any mental health morbidity among adults was found to be 7.5%. Therefore, the final sample size was calculated to be 3000, which was derived with a design effect of three, and an absolute error of 2% at the confidence level of 95%. The non-response rate was estimated to be 30%. Accounting for the work to be done and team size, 60 clusters of 50 adults were enrolled in the study.

### 2.4. Sampling Technique

**Figure 1:**
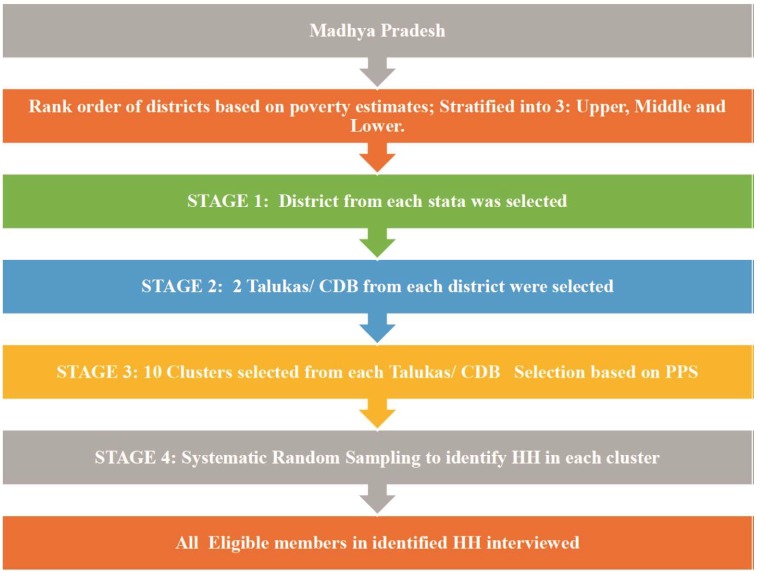
Sampling methodology adopted during the NMHS, 2015–2016.

The multistage, stratified, random cluster sampling technique, with random selection based on probability proportional to size at each stage (MSRS-PPS) was used. The cluster was either a named inhabited village or wards in an urban area as per the 2011 census. Cluster selection was done as per the PPS method. In each cluster, first, house listing and mapping were done; then, 15 households were selected by systematic random sampling. The primary and secondary sampling units were derived from the state’s districts and talukas. The districts were selected using district-level poverty estimates based on the stratified random sampling technique. Only non-institutionalized individuals were considered as a respondent. All of the resident members of the household (HH) were enlisted, and eligible members (aged > 18 years) were interviewed. In the event of the non-availability of a member of the household, two additional visits were planned. If the individual was not available even after three visits, the individual was declared as a non-responder. An informed consent was obtained from each respondent of the HH before initiation of the interview (Figure 1).

A qualitative methodology was adopted in order to explore the extent, pattern, and geographic distribution associated with substance/drug use and mental health problems, along with the community attributes such as stigma, health-seeking patterns, barriers, and challenges. Two focus group discussions (one each with patients and health care providers) and four key informant interviews (one each with a health care provider, social worker, health care provider from a Non-Governmental organization (NGO), and pharmacist) were conducted in each of the three selected districts.

### 2.5. Data Collection Tools and Procedures

A set of 08 study instruments was used in the study, which comprised:

1. Sociodemographic form: This form had information on age, gender, place of residence, income, education, occupation, and marital status.
2. Mini International Neuropsychiatric Inventory (MINI) 6.0 [23]: MINI was chosen for its multiple inherent advantages. It is an instrument that requires limited training to collect validated data, has validated translations in the Indian language, and could be administered to a large population. In epidemiological studies requiring psychiatric evaluation and outcome tracking, MINI is usually the interview of choice. With an administration time of fewer than 30 min, it is a short but accurate structured diagnostic psychiatric interview.
3. Intellectual disability: Intellectual disability, which was referred to as mental retardation in earlier times, has been included under the mental health program for programmatic purposes. Being a developmental disorder, it is not a mental health problem; however, because of comorbidities, overlaps still exist. The ID screener consisted of two questions, the response for which was recorded as either Yes or No, and probably yes was also recorded as Yes. A yes to any one of the two questions was considered to indicate positive ID. (1) Did the person appear backward, slow, dull, or markedly less intelligent in everything since childhood? (2) Did the person always have a difficulty in learning to do things that other individuals of his age did easily (for e.g., eating by oneself, dressing, bathing, toilet management).
4. Fagerström Nicotine Dependence Scale: This scale was used for tobacco assessment (smoking and non-smoking variants) [24].
5. Pathways Interview Schedule (Encounter Form): This World Health Organization (WHO) form was adopted and used to gather systematic information about the sources of care used by patients before approaching a mental health professional for assessing their health care-seeking behavior [25].
6. Sheehan Disability Scale: This scale was used to assess disability status and derive the related socioeconomic costs [23].
7. Assessment of Epilepsy: This assessment included questions related to epilepsy in order to provisionally diagnose generalized tonic–clonic seizures [26].
8. Socioeconomic impact on illness (modified as per WHO-Disability Assessment Schedule-2.0): This seven-question set was used to look at the subjective reporting of overall difficulties, the duration of these difficulties in the past 30 days, their impact on routine activities, expenditure due to illness, and whether a respondent was missing from family, social or leisure activities due to illness.

### 2.6. Training and Quality Control

The AIIMS Bhopal team in collaboration with the NIMHANS team conducted detailed training for the field survey staff (using a structured training protocol) for a period of six weeks that included theoretical orientation about the mental illnesses and survey methods, demonstration of asking questions to elicit mental illness symptoms, and supervised interviews in the hospital and later in the community. These trained field data collectors (FDC), who were postgraduates in psychology, collected data using hand-held devices that were configured for the purpose of the survey. Standard translation and back-translation protocols were used to translate all of the study instruments into the different local languages of the individual states. Daily and weekly monitoring and surprise on-field supervisory visits, along with systematic training of the field data collectors, ensured quality in data collection (including missing data). Quality of the collected data was also ensured by conducting re-interviews. Re-interviews were undertaken on 5% of the original interviews conducted by the field data collectors (FDCs). Re-interviews were conducted by the study investigators within one to two weeks of the actual interview by FDCs. Moderate agreement was observed between original and re-interviews, indicating that the quality of data obtained from the survey was quite reasonable and satisfactory, despite the limitations inherent to mental health disorders.

### 2.7. Statistical Analysis

Descriptive analysis provided estimates of the prevalence of mental illnesses coded by using the International Classification of Disease, 10th revision, Diagnostic Criteria for Research (ICD 10 DCR). The probability of selection and non-response was used to weigh the results. Analysis was done using SPSS 18.0 [27] and STATA 11.0 [28].

## 3. Results

A total of 3240 individuals were contacted and 2621 were interviewed during NMHS-MP (National Mental Health Survey-Madhya Pradesh) 2015–2016. An 80.9% response rate was achieved at the individual level, and 87.3% was achieved at the household level.

### 3.1. Mental Morbidity in Madhya Pradesh

The current prevalence of any mental illnesses amongst individuals aged >18 years was 13.9% (95% CI 13.7–14.1%), and the lifetime prevalence was 16.7% (95% CI 16.5–16.9%). Among current mental illnesses, common mental disorders (CMDs) including depression, anxiety, and substance abuse were reported in 13.55% of the individuals, whereas the weighted prevalence of severe mental disorders (SMDs) was 0.38%. The age and gender distribution of the study participants is shown in Table 1. Additional background characteristics of the study participants are provided in the supplementary materials (Tables S1–S4).

### 3.2. Treatment Patterns and Care Characteristics among Respondents with Current Mental Morbidity

There was a huge treatment gap of 90.7% among those identified with mental morbidity (n = 333) and those currently on treatment (n = 31). The median duration of illness was found to be 11 years, and the median duration of the treatment was five years. The median interval (in months) between onset of illness and consultation was found to be one year. Each patient consulted a median of two health care providers, and two-thirds of the time, the most recent consulted doctor was from the public sector (Table 2). Before accessing mental health care professionals, the majority of the patients admitted to seeking mental health care from temples, *dargahs* (shrines built over the grave of a revered religious figure, often a Sufi saint), local priests or traditional healers, and the main reasons cited for not seeking advice from the professionals were costly treatment, distant hospitals, lack of professionals, and unawareness of the availability of treatment (KII).

### 3.3. Substance Abuse Disorder in Madhya Pradesh

In this study, the prevalence of alcohol use disorder was found to be about 10.33% (CI: 10.19–10.46) and was much higher among male residents (20.23% CI: 19.98–20.49), those residing in urban non-metro areas (11.6% CI: 11.29–11.91), and those in the 40–49 year age group (16.07% CI: 15.69–16.45). Alcohol use disorder was found surprisingly to be the lowest among people living in urban metro areas (6.42% CI:5.93–6.91). The prevalence of tobacco use disorder was found to be the highest, i.e., 34.89% (CI: 34.68–35.1) and was highest among males aged more than 40 years and rural residents. *Khaini, Gutka, Nus, Bidi, Madhu,* and *Munnaka (Sasan)* were the most commonly consumed tobacco forms prevalent in local areas. Few health care workers also reported some unusual form of substance abuse such as cough syrups, drugs such as diclofenac, cetirizine, derivatives of barbiturates, whitener, and thinners, for which prevalence was found to be 0.6%. Stress relief, curiosity, recreation, lack of family support/emotional support, family conflicts for youths, and depression were some of the reasons mentioned by the participants for the consumption of these substances.

### 3.4. Suicide and Risk of Suicide

Suicidal risk was estimated to be present among 0.8% of the study participants. Males, residents of urban metro areas, and individuals aged between 30–49 years were found to be at the highest risk of suicide (Table 3).

### 3.5. Mental Health Services in Madhya Pradesh

Currently, the District Mental Health Program (DMHP) covers 14% of the total population of the state with a meager allocation of 0.2% of the total budget for mental health by the state health department. The state of Madhya Pradesh has two mental hospitals and 14 medical colleges with the Department of Psychiatry that are engaged in mental health care delivery services. About 12% of the district/general hospitals of the state are providing mental health services. However, only 3% and 0.1% of CHCs (Community Health Centre) and PHC (Primary Health Centre) respectively are providing mental health care services. Also, the number of core hospital-based mental health facilities in the state per lakh population was found to be near 0.03, whereas the numbers of beds available for mental health patient services was only 1.18 per one lakh population. It was found that only 124 health care professionals were available to deliver mental health services, with only two specialists and three trained MBBS (Bachelor of Medicine and Bachelor of Surgery) doctors for one lakh population. There are 0.2 mental health professionals and 0.05 psychiatrists in the state per one lakh population. There was also a shortage of rehabilitation workers and special education teachers in the state. Health care professionals who had undergone training in mental health in the previous three years were 99, i.e., 0.1 per lakh population.

### 3.6. Treatment Gap

**Figure 2:**
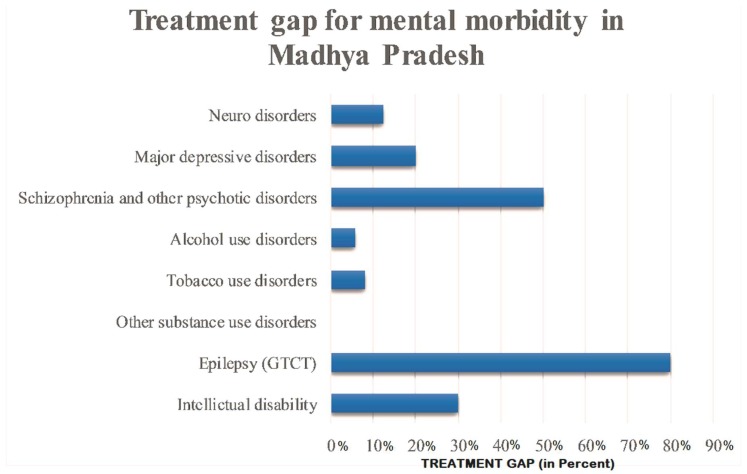
Treatment gap for mental morbidity in Madhya Pradesh.

The treatment gap for all mental health problems is as high as 91% in the state. Only 30% of patients with intellectual disability, 8% with tobacco use disorder, 5.8% with alcohol use disorder, 20% with major depressive disorder and 12.5% with neuro disorder received treatment. However, 80% of the patients with epilepsy received treatment (Figure 2).

### 3.7. Socioeconomic Impact of Mental Illnesses

**Figure 3:**
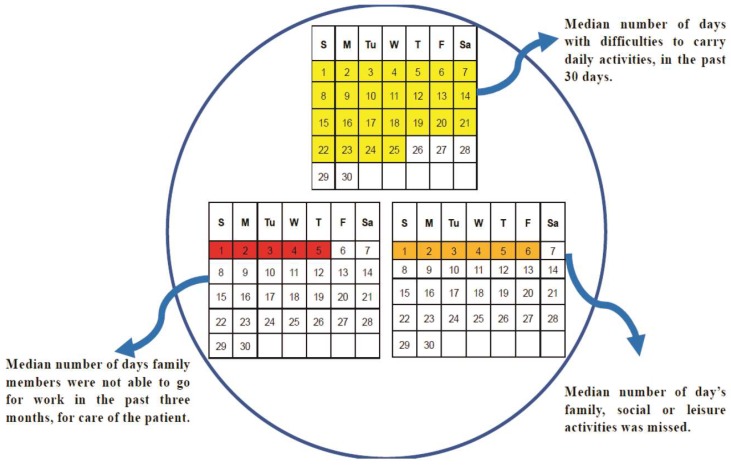
Socioeconomic impact of mental morbidities.

The median number of days in which difficulty was shown by people with mental morbidity in carrying out daily activities in the past 30 days was 25. Meanwhile, the median number of days for which the family members were not able to go for work due to the care of the patient in the past three months was five, whereas the median number of days where family could not attend family, social, or leisure activities due to the care of patient care was six, and the median monthly expense on the care of the patient was 1450 rupees INR (Figure 3).

## 4. Discussion

The present study is a part of the National Mental Health Survey of India, which was undertaken in 12 states of India. This is a first-of-a-kind comprehensive mental health assessment undertaken in the state of Madhya Pradesh performed on such a large scale. The strict quality control measures taken at multiple levels are the hallmark of this study, which provides reliable state-level estimates pertaining to mental health. The morbidity and treatment patterns along with the factors affecting mental health in Madhya Pradesh were unclear until now. This paper tries to report this crucial evidence, which is essential in order to provide better mental health care. The lifetime prevalence of mental morbidity and current mental disorders in the state of Madhya Pradesh is 16.7% and 13.5% respectively. This estimate is higher than the reported national average (13.9% and 10.5%) [17,21]. This estimate is also higher compared to other Indian states such as Gujrat (9.3% and 7.8%), Uttar Pradesh (8.7% and 6.6%), and Assam (8.1% and 6.0%), as well as other countries [29,30].

This study employed a mixed-method approach to capture insights into the patterns of mental morbidity and the treatment gap. The median duration of the mental illness was found to be 11 years with over half of the duration (six years) being left untreated. The treatment gap for mental disorders in Madhya Pradesh was found to be very high (91%) compared to the national average [17,21,31]. Affordability, accessibility, lack of awareness about availability of services, and a shortage of skilled mental health care professionals were among the common cited reasons for this gap. Also, it was interesting to find that individuals with mental health morbidity sought the help of alternative medicine (including traditional faith healers) before seeking modern medicine, which was consistent with findings from other studies [32,33]. Considering this, India may benefit from the integration of traditional healers into the service delivery of mental health care, as suggested by many studies done in Africa [34,35].

The prevalence of psychoactive substance use disorder is three times higher than the national estimate [21,22,36,37,38]. In recent studies by Chavan et al. [39] at Chandigarh and Gururaj et al. [40] at Bangalore, the prevalence rates were found to be lower than the present study. Among substance use disorders, tobacco use disorders are the highest in the state [21]; this is similar to the findings reported by Ghulam et al. [41]. Males aged over 40 years were the most affected due to tobacco use disorder, which is in conjunction with previous studies [42]. This high prevalence may be attributed to the social and cultural acceptance of tobacco consumption, as in some places, the local customs necessitate its consumption [21,22]. The suicide rate per lakh population in Madhya Pradesh is also found to be higher than the national suicide rate [21,22,43]. About 0.8% of people per lakh population were found to be at risk of suicide. The prevalence of suicide risk was found to be four times higher among urban metro dwellers as compared to individuals residing in a rural area. This can be attributed to the high-stress lifestyle that is characteristic of an urban metro area.

The availability of psychiatrists is least in Madhya Pradesh and has fallen short of the recommended requirement of at least one psychiatrist per lakh population, highlighting the severe inadequacy of mental health specialists in the state [44]. The lack of essential infrastructure and personnel needed for adequate mental healthcare delivery, including rehabilitative facilities such as daycare centers, halfway homes, sheltered workshops, temporary stay facilities, etc., can pose unique challenges.

Despite its high disease burden, mental morbidities and substance use disorder receive very little attention from the legislators and stakeholders of policy making in the state of Madhya Pradesh. Although mental morbidities are included in the existing routine Health Management and Information System (HMIS) in the state, the reality of the situation is not reflected, as the data related to this is scarce. Also, there is no separate budget head for mental health in the state. The total budget available for mental health was less than 1% in the state, and of the available budgetary support, utilization could not be done due to a lack of administrative and procedural clarity and skilled human resource constraints [45].

Thus, the development and proper implementation of policies for building a strong health system that integrates mental health with the larger public health system based on evidence-based practices is a burning need.

### Limitation of Study

The study does not include the children and adolescent population, which comprises a significant proportion of those with mental morbidities in the state.

## 5. Conclusion

This study has generated evidence of the high burden of mental disorders in Madhya Pradesh. It also highlights the huge treatment gap, which needs to be addressed as a priority. Despite this, the problem of mental health is neglected and left unaddressed during the planning and delivery of health care programs. Therefore, high priority should be ascertained for the development of inclusive and integrated mental health services, with a greater focus on substance use disorders.

## 6. Recommendations

The development of a standalone comprehensive mental health approach with specified goals and targets is the need of the hour. Being a high prevalent state in the context of substance use, the approach should be prioritized regarding the treatment of related morbidities along with a focus on the rehabilitation of the mentally unhealthy population and health-promoting activities. Quality-assured mental health services should be provided through the skill development of human resources engaged in mental health services along with an upgrade of the basic infrastructure and functioning of the concerned institution. Proper training of the existing manpower should be done periodically to enhance their basic expertise to deal with mental health issues both at community and facility levels. Apart from that, there is also a need to increase the involvement of qualified specialist viz. psychologists and psychiatrists along with the paramedical workforce so as to deal with the situation more effectively. Creating awareness among the upcoming younger generation through school-based Information Education Communication/Behavioral Change Communication (IEC/BCC) activities and the involvement of important stakeholders such as community leaders could boost the already existing preventive strategies.

More significantly, regular evaluation of the mental health services with a rapid and periodic appraisal of the programs should be done so as to stringently monitor the innovation, new actions/strategies, and their efficacy to deal with the mental health problems.

## Figures and Tables

**Table 1:** Age and gender distribution of study subjects (%).

Characteristics (*N* = 2621)	Proportion (%)
Gender	
Male	47.80%
Female	52.20%
Total	100.0%
Age Group (years)	
18–29	34.70%
30–39	22.90%
40–49	17.50%
50–59	12.20%
60–69	12.60%
Total	100.0%

**Table 2:** Treatment patterns and care characteristics among respondents with current mental morbidity.

Treatment-Related Characteristics (*N* = 333)	Frequency *
Currently on treatment (n)	31
Treatment gap (%)	90.69%
Median duration of illness (in months)	132 (1–480)
Median interval (in months) between onset of illness and consultation	12 (1–352)
Median number of treatment providers consulted	2 (1–10)
Most recent provider being a government doctor (n, %)	23 (74.19%)
Median duration of being on treatment (in months)	60 (1–480)

* The number in parenthesis indicates range, i.e., minimum–maximum.

**Table 3:** Prevalence of suicidal risk by age, gender, and residence. CI: confidence interval.

Classification	Biosocial Characteristic	Prevalence in % (CI)
Gender	Male	0.93 (0.87–0.99)
	Female	0.67 (0.62–0.72)
Residence	Rural	0.68 (0.64–0.72)
	Urban non-metro	0.83 (0.74–0.91)
	Urban metro	2.67 (2.35–3)

## Supplementary Materials

The following are available online at https://www.mdpi.com/2227-9032/7/2/53/s1, Table S1: Distribution of study subjects by marital status and gender (%), Table S2: Distribution of study subjects by education and gender (%), Table S3: Distribution of study subjects by occupation and gender (%), Table S4: Household Income (Quintiles).
