# Supplementary material for: Mental Health Issues in Madhya Pradesh: Insights from National Mental Health Survey of India 2016
Source: Healthcare (Basel). 2019 Mar 31;7(2):53. doi: 10.3390/healthcare7020053 (PMC6627290; doi:10.3390/healthcare7020053)
Supplement: Supplementary file 1 [file healthcare-07-00053-s001.pdf]

Miscellaneous tables:

Table 1: Distribution of study subjects by marital status and gender (%)

**Marital Status**	
Total (n)	**2621**
**Male**	**1254**
Never Married	19.6%
Married	78.4%
Widowed/Divorced/Separated	1.9%
Others	0.1%
Total	47.8%
**Female**	**1367**
Never Married	10.5%
Married	80.0%
Widowed/Divorced/Separated	9.4%
Others	0.1%
Total	52.2%
**Total**	**2621**
Never Married	14.9%
Married	79.2%
Widowed/Divorced/Separated	5.8%
Others	0.1%
Total	100.00%

Table 2: Distribution of study subjects by education and gender (%)

Education	
	**N= 2621**
**MALES**	**n= 1254**
Illiterate	23.00%
Primary	25.70%
Secondary	16.30%
High School	14.80%
Pre-University	9.30%
Vocational	2.10%
Graduate	6.00%
Post-Graduate	2.80%
**FEMALES**	**n= 1367**
Illiterate	47.80%
Primary	18.90%
Secondary	12.90%
High School	8.30%
Pre-University	5.30%
Vocational	0.30%
Graduate	4.30%
Post-Graduate	1.50%
**TOTAL**	**n= 2621**
Illiterate	35.90%
Primary	22.10%
Secondary	14.50%
High School	11.40%

Pre-University	7.20%
Vocational	1.10%
Graduate	5.10%
Post-Graduate	2.10%

Table 3: Distribution of study subjects by occupation and gender (%)

Characteristics	%
**Male**	**1254**
Cultivator	34.20%
Agricultural Labourer	20.80%
Employer	1.90%
Employee & Other workers	26.80%
Student	7.10%
Household duties	0.50%
Dependent	6.40%
Pensioner	0.70%
Others	1.60%
**Female**	**1367**
Cultivator	5.90%
Agricultural Labourer	12.20%
Employer	0.10%
Employee & Other workers	6.70%
Student	4.20%
Household duties	64.20%
Dependent	5.80%
Pensioner	0.50%
Others	0.20%
**Total**	**2621**
Cultivator	19.50%
Agricultural Labourer	16.30%
Employer	1.00%

Employee & Other workers	16.30%
Student	5.60%
Household duties	33.70%
Dependent	6.10%
Pensioner	0.60%
Others	0.90%

Table 4: Household Income (Quintiles)

Household income		
**Quintile**	**HH Income (Median)**	5000
**Q1**	**No of House holds**	176
	Min	0
	Max	2500
	Median	2000
**Q2**	**No of House holds**	169
	Min	2600
	Max	4000
	Median	3000
**Q3**	**No of House holds**	226
	Min	4100
	Max	6900
	Median	5000
**Q4**	**No of House holds**	170
	Min	7000
	Max	11500
	Median	9000
**Q5**	**No of House holds**	177
	Min	12000
	Max	150000
	Median	18500
